# Editorial: Non-coding RNA and Wnt/β-Catenin Signaling Pathway in Human Cancer

**DOI:** 10.3389/fphar.2022.837718

**Published:** 2022-04-21

**Authors:** Mary Miu Yee Waye, Weicheng Liang, Ernest Palomer, Zhong Liu

**Affiliations:** ^1^ Nethersole School of Nursing, The Chinese University of Hong Kong, Hong Kong, China; ^2^ Croucher Laboratory for Human Genomics, The Chinese University of Hong Kong, Hong Kong, China; ^3^ Biotherapy Center, The Third Affiliated Hospital of San Yat-sen University, Guangzhou, China; ^4^ Department of Cell and Developmental Biology, University College London, London, United Kingdom; ^5^ College of Life Science and Technology, Jinan University, Guangzhou, China

**Keywords:** non-coding RNA, Wnt/β-catenin, signaling pathway, circular RNA, mi-RNA, human cancer

**FIGURE 1:**
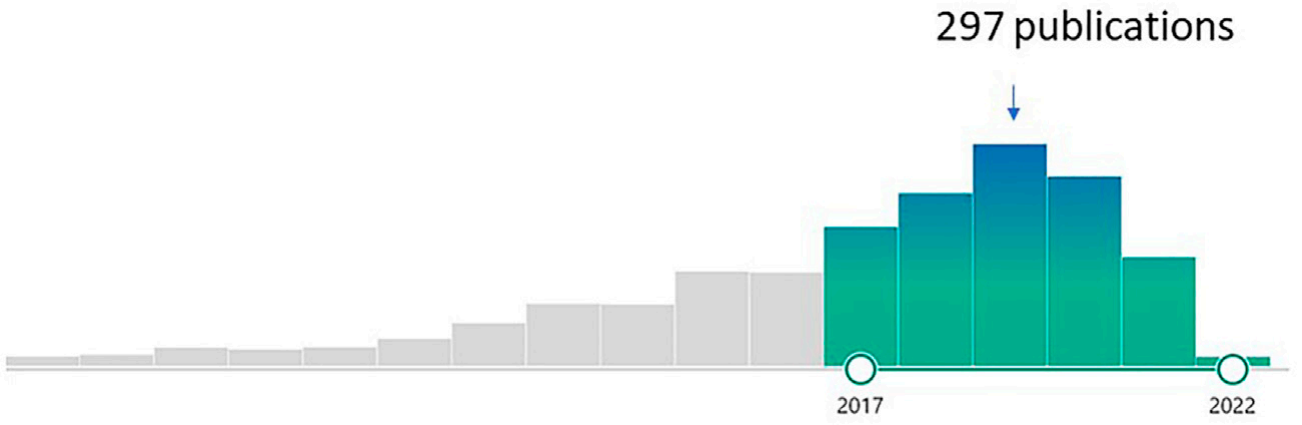
Trend of research on “Non-coding RNA and Wnt/β-Catenin Signaling Pathway in Human Cancer.” Green bars-highlighting the last 5 years’ publications (Source: Pubmed).

Our interest in Non-coding RNA and Wnt/β-catenin Signaling Pathway in Human Cancer were initiated from our earlier studies: one was that the long non-coding RNA (LncRNA-NEF) antagonized epithelial to mesenchymal transition and cancer metastasis via cis-regulating FOXA2 and inactivating Wnt/β-catenin signaling (Liang et al., 2018) and another one was that Lnc-H19 mediates methotrexate resistance in colorectal cancer through activating Wnt/β-catenin pathway (Wu et al., 2017). As the growing literature indicated that non-coding RNAs could orchestrate the Wnt pathway (Figure 1), Dr Weicheng Liang proposed to put forward a Frontiers research topic on non-coding RNA and Wnt/β-catenin Signaling Pathway in Human Cancer. Prof. Mary Miu Yee Waye rapidly agreed to contribute in this endeavour as it could shed light on the mechanism of cancer development and lead to novel drug development. Dr. Ernest Palomer and Dr. Zhong Liu joined the guest editorial board to broaden the overall background as they work on epigenetic regulation of Wnt signaling in the brain (Palomer et al., 2021) and in the role non-coding miR-150 in the proliferation and tumorigenicity in leukemia stem cells (Xu et al.) respectively.

Out of the many manuscripts that we have received, seven were published in this research topic, including 2 reviews and 5 original research articles.

One review contributed by Mu et al. summarized recent studies on the function and mechanisms of tumor resistance to cisplatin mediated by circular RNAs (circRNAs). The authors described various types of mechanisms in detail and the role of circRNAs in regulation of tumor proliferation, invasion, chemosensitivity, and other biological behaviors in the tumor microenvironment (TME). The authors emphasized that circRNA can be used as a promising target gene to reverse drug resistance and improve therapeutic efficacy.

A second review contributed by Li et al. summarized studies on the regulatory mechanisms of lncRNAs and their target gene signaling pathways in laryngeal squamous cell carcinoma, which is the second most frequent tumor of the respiratory system. This has significance in that by summarizing ncRNAs biological functions and important regulatory mechanisms in laryngeal squamous cell carcinoma the authors provide ideas for the improvement of diagnosis, prognostic evaluation, and development of pre-clinical targeted drugs.

The article contributed by Zhu et al. reported that the lncRNA LINC-PINT suppresses cell proliferation, invasion and Epithelial–Mesenchymal Transition (EMT) by blocking Wnt/β-catenin signaling in glioblastoma. They started with bioinformatics prediction of the role of LINC-PINT, followed by *in vitro* RT-PCR, clonal assays, and wound healing experiments to study the mechanism, and then they used *in vivo* tumor grating experiments to confirm the role of this lncRNA.

Liu et al. and colleagues reported that microRNA (miRNA)-142-3p inhibits tumorigenesis of colorectal cancer (CRC) *via* suppressing the activation of Wnt signaling by directly targeting β-catenin. They used clinical samples and compared miRNA expression profiles between healthy donors and CRC patients. Colony formation and MTT assays were used to test cell proliferation. Luciferase assay, immunohistochemistry, and Western blotting were employed to explore the molecular mechanisms.

The article contributed by Chen et al. reported that the lncRNA SNHG1 regulates the proliferation, apoptosis and autophagy of prostate cancer cells (PCa) *via* the Wnt/β-catenin and PI3K/AKT/mTOR signaling pathways. The PCa cells were transfected with a small interfering RNA plasmid (si-SNHG1) and si-SNHG1+multicellular protein EZH2 small interfering RNA plasmid (si-EZH2) to study the molecular mechanisms. Another article related to PCa was contributed by Jia et al. who reported that a traditional Chinese medicine Astragalus polysaccharides (APS) inhibits tumorigenesis and lipid metabolism through the miR-138-5p/SIRT1/SREBP1 pathway in prostate cancer. The approach used was microarray studies upon drug (APS) exposure, and they have successfully shown that ecoptic expression of SIRT1 inhibits the expression and nuclear translocation of SREBP1 *via* activating AMPK phosphorylation.

Shao et al. studied the anti-tumor mechanisms of curcumin in hepatocellular carcinoma (HCC). The authors reported a novel role for curcumin in inducing cell cycle arrest and apoptosis by downregulating the lncRNA LincROR, in turn reducing β-catenin and inactivating Wnt signaling. They approached the study by choosing lncRNAs that were previously reported to be related to tumorigenesis, and LincROR was the most down-regulated in the curcumin-treated HCC cells by examination of their expression levels.

Altogether, this research topic on non-coding RNA and Wnt/β-catenin Signaling Pathway in Human Cancer provided new ideas and mechanistic data on the role of several ncRNAs in cancers, shedding new light on the current state of the field and, more importantly, providing new avenues for future diagnostic and therapeutic avenues.
